# Perceived needs for attaining a ‘new normality’ after surviving myocardial infarction: A qualitative study of patients’ experience

**DOI:** 10.1080/13814788.2016.1274726

**Published:** 2017-03-03

**Authors:** Goranka Petriček, Josip Buljan, Gordana Prljević, Mladenka Vrcić-Keglević

**Affiliations:** ^a^ Department of Family Medicine, ‘Andrija Štampar’ School of Public Health, School of Medicine, University of ZagrebZagrebCroatia; ^b^ ’Zagreb Centar’ Health CentreZagrebCroatia; ^c^ Family Medicine Office Josip BuljanVelika KopanicaCroatia; ^d^ Family Medicine Office Gordana PrljevićKrapinske TopliceCroatia

**Keywords:** Qualitative study, myocardial infarction, patient’s experience, patient-centred care

## Abstract

**Background:** A comprehensive understanding of the various aspects of patients’ myocardial infarction (MI) experiences may help to guide these patients and their relatives through the many uncertainties they face and help them to stabilize their lives after the disruption they experienced.

**Objectives:** To explore MI patients’ experiences of life with MI, the challenges they face during the process of accepting their condition, and the setting and resetting of their personal goals.

**Methods:** Thirty semi-structured, individual interviews were conducted. The grounded theory method was used, and Atlas.ti qualitative data analysis software was used to facilitate the analysis.

**Results:** Three main themes and explanatory models emerged from the data analysis: a good adaptation – the ‘new normality;’ maladjustment – a continuous search for a ‘new normality;’ and perceived needs in the search for a new normality. Patients perceived several areas of need that they felt must be met before they could reach the state of a new normality. These needs included overcoming the anxiety of a possible MI recurrence; acquiring knowledge about MI in general and about ‘my MI’ in particular; the need for a timeline; for patience and steadiness; for both objective and subjective health status improvement; for taking control over the disease; and living within a supportive context.

**Conclusion:** When faced with a dramatic life event, most patients succeed in achieving a new normality in which they live changed but still satisfying lives. The needs experienced by patients when searching for a new normality may guide practitioners in leading patient-centred consultations.

KEY MESSAGES

- Most MI patients achieve a new normality.
- My physical identity is new: my body is different but still functional.
- My personal identity is new: I am not the same as before, the disease is part of me, but I retain parts of my previous self.

## Introduction

Myocardial infarction (MI) is an acute manifestation of coronary heart disease that can curtail both physical and psychosocial well-being and may thus adversely affect psychosocial adjustment in important life domains [1,2]. Faced with a growing population of aging patients who suffer chronic medical illness, the general practitioner (GP) must have strategies that accurately assess patient adjustment and facilitate effective coping [3]. The recent emphasis on adjustment to illness has shifted from a theoretical paradigm to the perspective of healthcare service provision and includes an interpretation of patients’ experiences within a ‘natural’ context [3–6].

This is the second paper that draws on a qualitative study by our group and investigates patients’ experiences of facing and living with MI about their healthcare needs and expectations. The analysis of patients’ initial experiences when confronted with a diagnosis of MI showed patients had varying needs for immediate emotional support and communication: patients experiencing an encounter with death expect a purely biomedical approach; in contrast, patients not facing a life-threatening situation expect immediate emotional support and the opportunity for their thoughts and concerns to be heard, explored and discussed [7].

In this second paper, we present the MI patients’ experiences after the initial, mostly hospital, phase. During this second phase, MI patients are mainly under the supervision of their personal GPs, so identification of factors and strategies that either help or compromise the adaptation process could be helpful to the GPs. Our aim was to find answers to the research question: ‘What are the patients’ needs and expectations in the process of adaptation and acceptance of MI?’

## Methods

### Study design

The study design presented in this article comes from the comprehensive qualitative study ‘Life with chronic disease: The patient’s experience,’ supported by the Croatian Ministry of Science, Education and Sports, registration number: 108–1080317–0280, and approved by the Ethical Board of the Zagreb University School of Medicine (Number 04–1162–2006).

The methodology has been explained in detail elsewhere, so will be presented only briefly [7,8]. Semi-structured individual interviews were performed to collect data and the interviews were transcribed verbatim. Grounded theory was used for data analysis and interpretation [9,10]. The investigator triangulation method was used during analysis to check the qualitative research validity [7,11].

### Selection of study subjects

The study was conducted in the general practice setting, and 16 GPs were chosen to recruit the patients, using a maximum variation sampling strategy [11]. These GPs recruited 32 patients with a primary diagnosis of MI by applying theoretical sampling strategy [12]. In the first phase, eight GPs chose one male and eight GPs one female patient, according to predefined inclusion criteria [7,13]. In the second phase, a further 16 patients were recruited according to the results of iterative data analyses to explore analytically relevant distinctions [10,12].

### Data collection

The first author carried out semi-structured, individual interviews. The tape-recorded interviews, guided by an interview-guide, were transcribed verbatim. From the 32 transcripts, 30 were used for the analysis, as two failed to meet Kvale’s quality assurance criteria [13].

### Data analysis

The theoretical framework was developed and refined by applying open, axial, and selective-coding procedures to every transcript. The main investigator initially did the analysis by using the Atlas.ti analytic tool [14]. In parallel, a group of three investigators individually analysed all 30 interviews manually. Next, these three met to discuss the results and reach consensus. Finally, the main investigator compared her results with those of the group. The results represent consensus among all four investigators.

## Results

Thirty patients, 16 men and 14 women were interviewed. The average age was 53.6 ± 6.4 (SD). The average time that had passed since their MI diagnosis was 3.7 ± 1.3 years. In addition to MI, all patients reported one or more co-morbidities, the most frequent was hypertriglyceridemia (all), followed by hypertension (24/30), and diabetes mellitus type 2 (18/30).

Although each patient’s account of their adaptation experience was unique and highly contextual, three main themes and explanatory models emerged from the data analysis: good adjustment—the ‘new normality’; maladjustment—a continuous search for the ‘new normality’; perceived needs in searching for the ‘new normality’.

### *A good adaptation*—*the ‘new normality’*

According to the patients’ experience, the disease was fully accepted when it became ‘just a normal part of life, just another daily duty to think about and deal with’ (F8) (Box 1). To establish this new normality (F8), patients needed to recognize that they and their bodies, while different, were still functional. They had lost something of their previous life, but at the same time, they had gained some benefits. The patients stressed the importance of not losing their self-identity; they needed to retain important values and characteristics of their previous selves. They also expressed the importance of gaining self-esteem by finding a balance between themselves and their disease, and of gaining the self-awareness that they can take control of both the disease and their own lives. One patient said, ‘Crucial to that was my realization that I can altogether control it’ (F9). However, they acknowledged that sometimes they were in command of the disease and sometimes the disease took control. They learned to accept the disease as a ‘part of me’ (F6), or as a life partner (M10). ‘If we (me and my MI) get along well, everything else goes well,’ one patient said (M2). Many patients stressed that being accepted as a ‘complete person’ (F1), still needed and useful to loved ones, and also returning to work provided important confirmation of their new normality.

**Box 1.** A good adjustment—the ‘new normality’

Disease is just a normal part of my life, just another obligation to think about	‘Well, I accepted the disease when I accepted its rules…. There is a certain regime and certain restrictions. Moreover, the regime of life must be matched accordingly…. Thus, the disease becomes the headquarters of importance, and the person; in fact, he does not lose anything, but should just be aware of: do this, do that, can do this … so that person needs to adjust to it! The disease is a significant element which should be considered and according to which men should harmonize their activities!’ (M12) ‘The disease is just a normal part of life, just another daily duty to think about and deal with.’ (F8) ‘The disease is there, but I’m not upset about the disease, it is part of my life … ‘ (M6) ‘To acclimatize to the disease … just another daily concern … you’re taking medication … you think about food … physical activity.’ (M4)
My body is different but still functional	‘I realize that I cannot live like before, and I can only do things to the extent my chest pain permits! … And it was only a year after my heart attack that I realized that I had had a heart attack and had to take some medication and exercise until the end of my life, but it does not matter, I can still live, function, enjoy life…’ (F12) ‘I can never be as I was before. Because the years make their impact and disease makes its impact. But I don’t see any problem.’ (M10) ‘I had to arrange, to adapt, to modify … and now I can function so that pleases me … I’m less mobile, but I’m not disabled … I don’t consider myself disabled.’ (F5) ‘Now I listen to my body… when my body lets me I walk, if not, it does not matter … I will do it tomorrow…’ (M13) ‘I used to be impatient, lived full throttle … now I reduce the speed of … and everything goes well …’ (M4) ‘Then I accepted MI, when I saw that there is hope and there is life … different … but I can live with it.…’ (M5)
I managed to keep something valuable from my ‘past’ life	‘I accepted the disease when I completely learned to live with it, when I realized that the disease was part of me, that I can handle, cope with it—my new normality! I have learned to recognize when I overdo it with my other life activities and have to stop for a bit. I need to take medication, I need to be careful but I can still live a satisfying life.’ (F8) ‘… my old cottage, …. Whenever I go there … I just touch the meaning of my life …’ (M4) ‘I have land with more than 60 types of flowers.… This is what I love, what makes me happy!’ (F5)
I even gained some benefits	‘Many good things happened! I quit smoking, firstly reduced, and then completely stopped. Then my wife and I started hiking; in the beginning, we just went for a walk and now we are regularly going every weekend! Because of my disease, we as a family became stronger. Somehow, we started to be more strongly attached and we rely on each other much more. There I see even the "plus" of my disease!’ (M2) ‘My family helps me more. Before MI, it was normal that certain things I do, in fact all the housework (laugh). Now they have taken over some of those things. That is important to me. That pleases me.’ (F1) ‘Disease helped me to control my nerves. It is as if the disease has opened my eyes in some sense. I think its better that it (MI) hit me in time when change could still have an effect. Now I do not get upset so much; I just calm down. And I feel much better.’ (M8) ‘My son has become a good student … as if this disease encouraged him!’ (F6)
I gained self-esteem by creating a balance between myselfand the disease	‘To accept the disease means to live in harmony with it, but again I say to some extent! Because I am not the type who will blindly adhere to some sort of ban. I’m in the horoscope Aquarius—freelancer, my character is like that!’ (F5)
Taking control of my life	‘Yes, I accepted my disease. Crucial to that was my realization that I can altogether control it, that much depends on me. That single moment when I realized I could deal with my disease, encouraged me strongly!’ (F9) ‘I would say that I accepted the disease but I am still in charge of my life.’ (F1)
Disease is my life partner	‘If I don’t respect my disease, the disease wouldn’t go as expected; it could go in the wrong direction! Therefore, the disease is my life partner. If we get along well, everything continues going well. However, if we start a fight, for example, if I decide not to follow the treatment recommendations, then I know that my blood pressure, sugar, and fat will be high. Also, if I do as is recommended then all is good. Therefore, I consider it every time. But at the same time I am OK, I am not afraid.’ (M10)

### Maladjustment—a continuous search for the ‘new normality’

Very few patients indicated that the quest for their ‘new normality’ was ongoing (Box 2). For them, MI remained an ‘imaginary’ construct, not an everyday reality. Sometimes, the disease was completely forgotten and sometimes it was ‘hard to tolerate’ (F7), and the idea of living with MI made them extremely anxious. The occurrence of any symptom sent them back to the beginning of the adjustment process. Feeling they were losing control over their lives contributed to their maladjustment. Some of these patients stubbornly resisted the disease and its consequences, as they did not want to be dependent on the disease or to become a burden on their relatives. They stated that taking medication and the restrictions imposed by the illness were everyday reminders that they were sick.

**Box 2.** Maladjustment—a continuous search for the ‘new normality’

Struggling against MI	‘And when you ask me whether I accepted my disease: I didn’t … still keep up fighting with it! I keep telling myself: “I’m not sick, it’s not a disease!” And even now, I always feel better when I visit my little cottage … simply forget what my doctor says…’ (M11)
MI makes me anxious	‘Whenever I feel chest pain I start to think about the disease and realize that I’m still sick, that I’m going to die, and I become sad, anxious, thinking this is the end…. Every single day that struggle…. I even used to go to meditation … to learn how to deal with it, to calm myself. For nothing … so, whenever I feel the pain, it almost immediately pulls me back to the beginning of my battle… in only one moment I am at the beginning again.’ (F7) ‘For such a long time I was so quite shaken by the disease! In fact, I am often still shaken…. Actually, I have not yet fully accepted this disease. I am still holding a certain fear… ‘ (F13)
It’s just abnormality in diagnostic tests	‘Well, I didn’t felt my MI at all, no pains like others. OK they said, my ECG showed that my MI happened before. Nevertheless, this was only a changeable deviation, similar to a situation when my blood sugar was high, so I ate less and walked more … my blood sugar improved. Therefore, I live my life as before. Although, from time to time I get angry, and eventually land in some depression…’ (M15)

### Perceived needs in the search for a ‘new normality’

Several of the patients’ perceived needs were identified in the search for the ‘new normality’ (Box 3).

**Box 3.** Perceived needs in the search for the ‘new normality’

Overcoming the anxiety of MI recurrence	‘At first … I was very tired and often felt stabbing chest pains. I would keep silent, terrified, thinking: ‘Shall I die? Is that MI again?’ I could not even move my body. (F1) ‘Well, fear influenced me not to accept my disease immediately … instead of making an effort to change things, I was afraid of the present and the future with the disease … as if it blocked me!’ (M7)
Need for information	**Need for information—about MI in general** ‘Knowing about the disease really calmed me, helped me to relax…. I had to educate myself about the disease, read a lot and learn about it … what caused it, the possible course and consequences, how to behave … I think one has to know a lot! For instance, I learned that sugar and fat clog blood vessels and cause a heart attack. It’s important to know what will help, so you know how to behave.’ (F13)
	**Need for information—about ‘my MI’ in particular** ‘I don’t know why that myocardial infarction struck me. It drives me crazy! I have no pressure, no sugar, and no fat, nothing…. Everyone says it’s probably stress …’ (M12) ‘Until I became well acquainted my disease, I could not move on … and constantly living with it was tremendous pressure! I did not know how to interpret the symptoms. How long would it last? What was next? That was my biggest problem! Initially, I was extremely afraid; I did not dare get out of bed…. when I tried to walk I often felt stabbing here (the chest) … and I immediately thought: “Here we go again, I’m done for! It is re-MI!” So I often ended up in an emergency ward unnecessarily.’ (M8) ‘With time, I learned to recognize that pain, and learned a lot about my disease, why does it hurt, and how to help myself.’ (F1) ‘With time, I learned to recognize, from my experience, if a chest pain comes from my heart, or from another unimportant organ, or if I am just anxious! So I can orient myself to whether it is serious or not.’ (M3)
Timeline	**The lapse of time** ‘I knew I needed time to get everything in place … and indeed that time has come.’ (M14) ‘It took time to settle down … but as they say—in the end time cures all.’ (F4) ‘You have to consider with yourself … to think … to realize: Now this is allowed and that is not good to do … you have to get used to it … look into your own eyes.’ (M2)
	**Time in daily life** ‘My husband, children… I did not even have time for myself, for my disease… so it took me a long time to settle down.’ (F6)
Patience and steadiness	**Patience and steadiness** ‘So, I gradually, little by little, got accustomed (to MI), and calmed down. And so… over time I realized that lots of it depend on me, that I can control things to a greater extent… little by little…’ (M13) ‘My husband said, ‘Let’s go hiking again!’ In the beginning it was very difficult, but slowly, slowly and with great effort, I returned to the old and yet a new me.’ (F8)
	**Struggle within himself/herself** ‘You have to accept your disease such as it is. It was hard in the beginning, but you should try, and keep trying, even if you do not have results yet! All those recommendations about diet and exercise… And the first few times I tried to go through the wall, but then I stopped… I simply couldn’t otherwise.’ (M16)
	**Playing a hide and seek game** ‘In the beginning, I often acted as if nothing had happened. This diagnosis was like a shackle around my neck and it will be with me ‘till the end! And if so, I do not need always to be mindful of it! I felt I would be at least somewhat free if I did not follow all those recommendations. Just to breathe easier! Therefore, I did not always keep to the treatment, and played hide and seek, at the same time, feeling normal as I did before MI. But soon, I realized I was not cheating the physicians, I was cheating myself.’ (F1) ‘I had just taken drugs at the pharmacy but never been taking them really—I simply left them in the drawer … until I got sick again…. Thank God I had those drugs at home then.’ (F2)
Objective and subjective health state improvement	**Positive experience with initial attempts** ‘Within a week I wanted to start walking, but somehow I didn’t have the strength. I tried a little bit up the hill, but it did not work. Therefore, I tried again and I slowly climbed the first step, then another day two steps, and the third day I did halfway up the stairs! I was looking forward to any progress! Progress pushes me still further!’ (M5)
	**Improved results from the diagnostic tests** ‘I immediately accepted my disease after they gave me my coronary angiography findings! They (the physicians) had done it all so beautifully! It also helped when finally everything started to get better: blood pressure, blood sugar, fat!’ (F6)
	**Having a subjective feeling of good health** ‘The fact that I felt well, soon after the heart attack, made my adjustment easier! I felt well and that was a basis for hope, but again, on the other side, was the caution that something unscheduled could happen….’ (M14)
Taking control over the disease	‘So it took me about a year and a half to adjust to the disease, to realize that disease is an inseparable part of me, but something that I can handle. I gained the ability to realize when I have to slow down, if I overdid it with my business or family activities, that I needed to think about the recommendations….’ (F8)
Social network support	‘My family was with me from the beginning! Completely. My husband and kids and mom and my brother and sister…. And it was very important for me, not only as an emotional and practical support, but they were one of the reasons why I am well-adjusted and still moving on….’ (F9) ‘After the MI, the family started to look at me a little differently … like I am sick, not as a healthy person, but … sometimes…. Sometimes it bothers me when they advise me to be careful—“You can’t do this, you can’t do that!” They look at me like a drop of water on the palm—I simply do not like it! I would rather be seen as a normal healthy person, it doesn’t suit me to be looked at as a patient!’ (M10)

#### Overcoming recurrence of MI anxiety

Patients reported that after MI life was imbued with uncertainty. They stressed their anxiety over a possible recurrence as one of the most negative emotions they had to face, especially during early recovery. This anxiety increased with the onset of various chest symptoms and sensations (a type of stabbing and compression), which appeared during patients’ initial attempts to restore everyday activities, especially physical activities. Their anxiety limited and even blocked their initial attempts at adaptation. Overcoming this anxiety was the starting point in the process of adjustment.

#### Need for information—about MI in general and ‘my MI’ in particular

Patients emphasized that information gathering helped their stabilization and positively affected adjustment and coping with MI. They expressed two types of information needs. The first was a need for knowledge about MI in general, including causes, disease presentation, course, possible complications, treatment, and self-help procedures. The second type of information needed was for specific knowledge about ‘my disease’—‘my MI’ in particular. They expressed the need for self-reflection asking, ‘What was I doing wrong?’ They needed to become familiar with their ‘new physical body’, with the symptoms, when they appear and disappear, how to prevent or reduce them, and especially how to distinguish symptoms associated with MI from those with other causes.

#### Timeline

The need for time was experienced in two areas. First was the importance of the lapse of time: as time passed, patients learned to live with their disease (from the cognitive and emotional perspectives). ‘Time cures all,’ they said. Patients also emphasized the importance of having enough time in their everyday lives for self-reflection, cognitive reconciliation of themselves with their disease and for emotional acceptance of a new normality. The lack of time in everyday life was often highlighted as an aggravating factor, especially in those still carrying active and irreplaceable life roles.

#### Patience and steadiness

The adaptation process does not follow a straight line. For the patients, it was a tortuous path of trial and error, two steps forward and one step back. Then they realized, as one patient put it, ‘I was not cheating the physicians I was cheating myself’ (F1). Patience and steadiness were needed from patients and the persons surrounding him/her.

#### Objective and subjective health state improvement

Patients expressed the need to see positive results from their previous attempts in various activities, including feeling good after physical exercise and the positive results after adhering to treatment. Patients needed tangible forms of encouragement, such as improved results from diagnostics test. In addition to the objective parameters, the subjective feeling of good health was an important and empowering factor during the adjustment process.

#### Taking control over the disease

Patients expressed the awareness that ‘my capability’ for gaining control over the disease was an important aspect of their experience.

#### Social network support

Most of the patients emphasized the need for support, first from family and friends, then from health professionals. However, they mentioned the negative impact of a ‘too protective’ attitude: they still needed to be ‘master’ of their lives.

## Discussion

### Main findings

The study results show that after a few years most of MI patients achieve a state of good adjustment to their MI. During this process, their state of normality changes: they report a new personal identity. Patients differ in the way they perceive themselves, some adapt well to a ‘new normality,’ whereas others experience ‘maladjustment with a continuous search for that new normality.’ The study yielded additional knowledge on the needs patients face. First, they need time, not only a lapse of time, but daily time for self-reflection and reconciliation. Lack of time in everyday life is an aggravating factor, especially in those still carrying active and irreplaceable life roles. Second, the adaptation process is not a straight line; it is a tortuous path of trial and error. Therefore, patience and steadiness are needed from the patients as well as from the people around, including their GPs. Third, they need knowledge about MI generally, and about the specific characteristics of ‘my MI.’ The meeting of these needs helps to overcome anxiety about the recurrence of MI and assists patients in achieving a new normality.

### Strengths and limitations

The strengths and limitations of the study design were discussed in our previous article [7]. We believe that its strength comes from its descriptive and narrative framework in which the patients’ perceived needs in searching for the new normality are elaborated on from a practical perspective so they can be integrated into the daily work of GPs [15].

The main limitation may come from the fact that the patients’ experiences were examined two to five years after the onset of MI. There is a general assumption that most people will internalize the disease within two years but we are not sure if other themes would arise with a further lapse of time [16]. Our participants came from a broad range of socioeconomic backgrounds; but it is possible that our results would not be completely applicable to other localities, ethnicities and religious and cultural groups [17,18].

### Interpretation in relation to existing literature

Consistent with previous findings, most patients in this study achieved a state of good MI adjustment and were able to regain control over their lives [19–24]. These findings are consistent with the health belief model (HBM) [25] and the common sense model of self-regulation [26], theoretical models often used to understand the behaviour of patients with MI [25]. The behaviour of the patients in our study correlated with perceived MI severity and the perceived benefits and barriers when making the changes, which fits with the HBM. As suggested by the common sense model, our patients were active problem solvers; they formed and activated plans for coping with MI and their emotions, and they evaluated the success of their actions. When necessary, their coping plans were reassessed and changed [26,27]. Insight into the positive aspects of their ‘new lives’ was helpful and, as suggested by Petrie et al., prompted healthier lifestyle choices, greater appreciation of health and life, improved close relationships and changes in personal priorities [24–27]. While the literature shows that the adjustment can be prolonged and is unsuccessful in approximately 30% of patients, very few patients in our study had difficulty in accepting their MI [28].

Of course, it is not possible to draw comparisons with the results of quantitative studies, but maladjustment deserves attention because it is often equated with psychological morbidities, such as depression, anxiety, distress or behaviour problems [28,29]. While still learning to live with MI, our patients often ‘cheated’—acting as though nothing had happened. According to the patients, this gave them some sense of control over their lives [18]. Positive previous experiences in attempting to make changes, as well as improved results from diagnostic tests, were empowering factors in facilitating adaptation [27,28]. Consistent with previous findings, this study found that a close social network affected recovery positively, but that some patients experienced over-protection as an obstacle [24,27,28]. Although, Bergman and Bertero found that some patients with acute coronary syndrome were often unwilling to return to full-time work [29], in our study returning to work was perceived as a sign of good adjustment and a new normality.

Few studies on the experience of MI patients are based on grounded theory. Applying it as the conceptual framework in this study allowed us to understand key processes in health behaviour from the MI patients’ perspectives. It enabled us to identify and explain the behaviour of MI patients [30]. This knowledge enabled us to get alongside the patient’s needs and expectations during their process of adaptation to MI.

### Implications for clinical practice and research

GPs could use the study results as guidelines when exploring the needs of a particular MI patient (Box 4). We believe this approach might prove helpful in communicating with a patient’s family, facilitating early discussions about the challenges the patient is facing as well as the needs at each stage of the illness. This is important since early involvement of the patient’s family is essential for a positive outcome [31]. Patients’ stories have also been helpful in small-group discussions, and we have used them when working with medical students and GP trainees. Again, since each patient’s experience is unique and deeply embedded in the context of his or her life, there is a need for future research in different population groups and different settings. This research is even more important for GPs today as they work in global societies enriched by cultural diversity.

**Box 4.** A guideline for GP in exploring and addressing the specific needs of an MI patient within the patient-centred consultation.

**Topics for discussion with patients**
Overcoming MI recurrence anxiety
The need for information about MI
The need for information about his/her MI
The need to understand a present situation
Positive experience with initial attempts
Improved results from the diagnostic tests
Subjective feeling of good health
Balance between him/herself and the disease
Control over the disease
Social network

## Conclusion

After the onset of MI, most of the patients in this study reached a state of ‘new normality’—a good MI adjustment—while very few patients remained in a state of maladjustment. In the process of searching for this new normality, patients identified several needs: to overcome their anxiety about the recurrence of MI, to acquire knowledge about MI in general and about ‘my MI’ in particular, and to reach both an objective and subjective health status improvement. Two aspects of time—the lapse of time and having enough daily time—are needed, while the patients need steadiness, both from within themselves and also from the people surrounding them, if they are to pass successfully through the uncertainties they face. Taking control over their disease and being in a supportive context both contributed to the establishment of the ‘new normality’.
